# The role of the STAS domain in SLC26A9 for chloride ion transporter function

**DOI:** 10.1016/j.bpj.2024.05.018

**Published:** 2024-05-21

**Authors:** Satoshi Omori, Yuya Hanazono, Hafumi Nishi, Kengo Kinoshita

**Affiliations:** 1 Graduate School of Information Sciences, Tohoku University, Sendai, Miyagi, Japan; 2 Department of Bioscience, Nagahama Institute of Bio-Science and Technology, Nagahama, Shiga, Japan; 3 Medical Research Institute, Tokyo Medical and Dental University, Bunkyo-ku, Tokyo, Japan; 4 Faculty of Core Research, Ochanomizu University, Bunkyo-ku, Tokyo, Japan; 5 Tohoku Medical Megabank Organization, Tohoku University, Sendai, Miyagi, Japan; 6 Institute of Development, Aging, and Cancer, Tohoku University, Sendai, Miyagi, Japan

## Abstract

The anion exchanger solute carrier family 26 (SLC26)A9, consisting of the transmembrane (TM) domain and the cytoplasmic STAS domain, plays an essential role in regulating chloride transport across cell membranes. Recent studies have indicated that C-terminal helices block the entrance of the putative ion transport pathway. However, the precise functions of the STAS domain and C-terminal helix, as well as the underlying molecular mechanisms governing the transport process, remain poorly understood. In this study, we performed molecular dynamics simulations of three distinct models of human SLC26A9, full-length, STAS domain removal (ΔSTAS), and C-terminus removal (ΔC), to investigate their conformational dynamics and ion-binding properties. Stable binding of ions to the binding sites was exclusively observed in the ΔC model in these simulations. Comparing the full-length and ΔC simulations, the ΔC model displayed enhanced motion of the STAS domain. Furthermore, comparing the ΔSTAS and ΔC simulations, the ΔSTAS simulation failed to exhibit stable ion bindings to the sites despite the absence of the C-terminus blocking the ion transmission pathway in both systems. These results suggest that the removal of the C-terminus not only unblocks the access of ions to the permeation pathway but also triggers STAS domain motion, gating the TM domain to promote ions’ entry into their binding site. Further analysis revealed that the asymmetric motion of the STAS domain leads to the expansion of the ion permeation pathway within the TM domain, resulting in the stiffening of the flexible TM12 helix near the ion-binding site. This structural change in the TM12 helix stabilizes chloride ion binding, which is essential for SLC26A9’s alternate-access mechanism. Overall, our study provides new insights into the molecular mechanisms of SLC26A9 transport and may pave the way for the development of novel treatments for diseases associated with dysregulated ion transport.

## Significance

We explored the mechanism by which the human protein SLC26A9 transports chloride in the cell. SLC26A9 is a potential therapeutic target for patients with cystic fibrosis, as by targeting drugs to it, it may be possible to restore chloride ion transport in epithelial cells. To design therapeutic drugs, it is essential to understand how the protein works. Our findings support an alternate-access mechanism in which chloride ions bind to SLC26A9 inside the cell and are then released by the protein to the extracellular environment. We find that the STAS domain of SLC26A9 has critical roles in binding chloride and induces conformational changes in the transmembrane domain that facilitate chloride transport.

## Introduction

Chloride ions, the most abundant anions in the extracellular fluid, are involved in a variety of physiological processes, such as the regulation of cellular pH, the control of membrane excitability of nerve or muscle cells, and epithelial transport (1,2,3,4). The dysfunction of chloride ion transport results in diverse disorders, including epilepsy, myotonic disorders, and cystic fibrosis (CF). CF is a genetic disorder caused by mutations in a chloride channel, CF transmembrane conductance regulator (CFTR) (5,6,7). Due to a low transport capacity for chloride ions, sticky secretions clog the bronchial and digestive tracts, leading to diseases such as pneumonia and bronchitis. Though CF is a serious disease frequently seen in Caucasians, there is no cure for CF at present, although there are therapeutics directed to CFTR with some efficacy, depending on the underlying mutation.

SLC26A9, which is a member of the solute carrier family 26 (SLC26) of anion transporter/channel proteins, is mainly expressed in the lung and gastric epithelium and contributes to mucociliary clearance and gastric acid production (8,9,10,11). SLC26A9 is regarded as a chloride ion channel or an uncoupled fast chloride transporter with channel-like activity (12,13,14,15,16). On the other hand, it is also reported as a bicarbonate/chloride ion exchanger (17,18,19) or a sodium/anion cotransporter (18). These insights suggested that this difference in transport mechanism could be because of the cell and tissue context (8,10).

SLC26A9 interacts with CFTR to regulate chloride ion conductance (14,20). Variants of SLC26A9 are associated with CF-like lung disease (21,22) and modulate the response of the airways to CFTR-directed therapeutics (23,24). Given their colocalization and the functional correlation between SLC26A9 and CFTR, SLC26A9 is a potential therapeutic target for patients with CF to restore chloride ion transport in epithelial cells.

**Figure 1:**
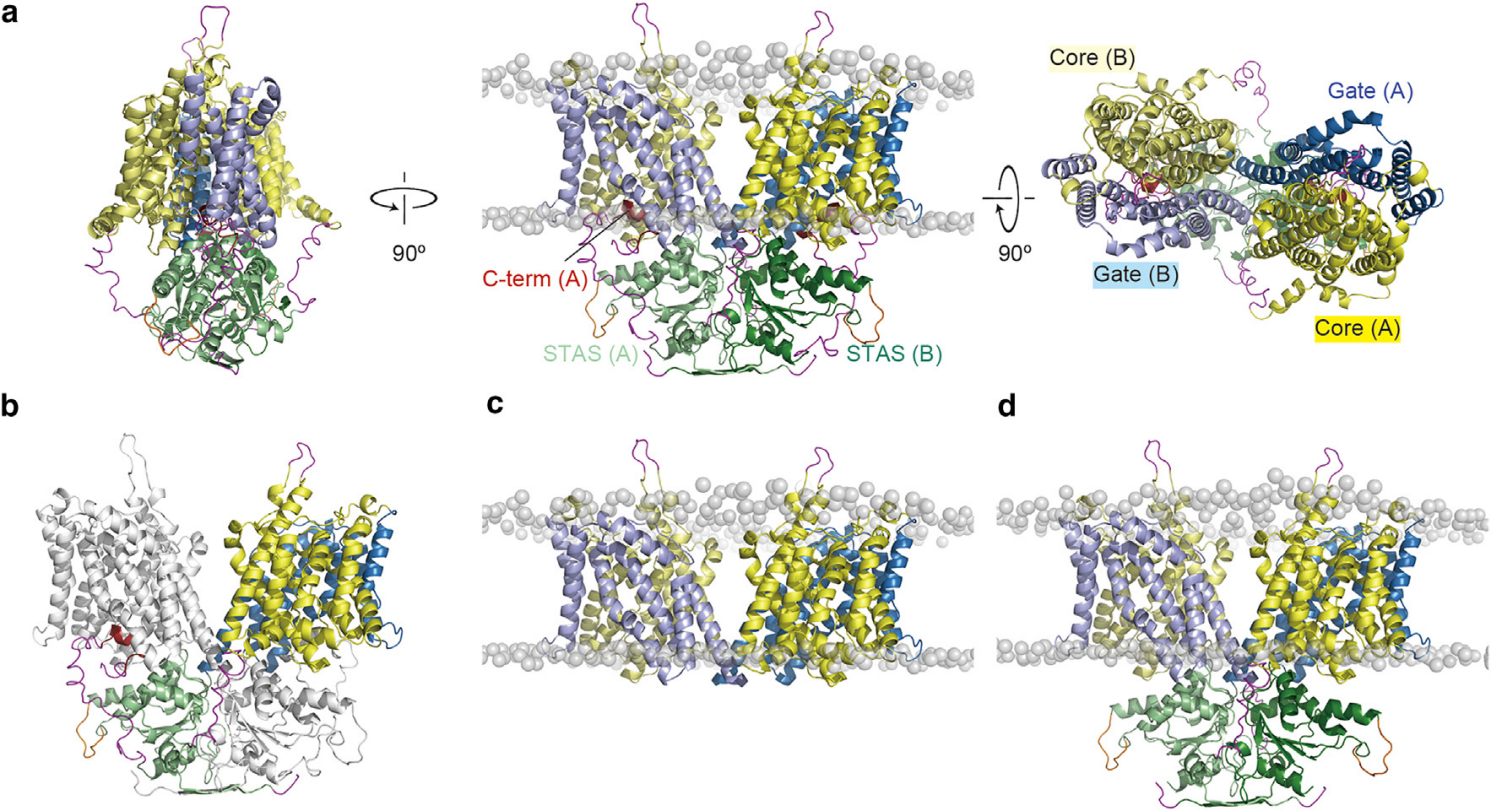
Initial structures of (*a* and *b*) FL, (*c*) ΔSTAS, and (*d*) ΔC simulations. Cartoon representations of the core (residues 45–150, 158–198, and 313–428, *yellow*), gate (residues 199–312 and 429–499, *sky blue*), and STAS (residues 5–26, 500–567, and 653–741, *light green*) of chain A and the core (residues 45–150, 158–198, and 313–428, *pale yellow*), gate (residues 199–312 and 429–499, *light blue*), and STAS (residues 5–26, 500–567, and 653–741, *deep green*) of chain B are shown. C-terminal ordered regions (residues 773–783, *dark red*), the modeled loops of the truncated portion (residues 568–573 and 648–652, *orange*), and the short disordered regions (residues 1–4, 27–44, 151–157, 742–772, and 784–791, *magenta*) are also shown. Residue numbers are based on the cryo-EM structure of SLC26A9 (PDB: 7CH1). Phosphorus atoms in the headgroups of lipid membranes are shown as gray transparent spheres. (*b*) Chain B is colored white to show the characteristic X-shaped dimer structure. To see this figure in color, go online.

SLC26 proteins belong to the sulfate permease family that is conserved in various species from bacteria to mammal (25,26). The sulfate permease family proteins consist of two domains: an alpha-helical transmembrane (TM) domain and an STAS domain located in the cytoplasmic region. The topology of the TM region consists of 14 TM helices characterized by 7+7 inverted repeat folds (27,28,29). Recently, several high-resolution structures of SLC26A5 (30,31,32) and SLC26A9 (33,34) have been reported using cryoelectron microscopy (cryo-EM). The structures of mouse (33) and human (34) SLC26A9 were determined at 3.9 and 2.6 Å resolution, respectively. The cryo-EM structure of mouse SLC26A9 was performed using a truncated variant lacking the intrinsically disordered region of the long intervening loop in the STAS domain and C-terminus. The cytoplasmic STAS domain is important for forming a homodimeric structure and acts as a platform for the interaction of subunits. Dimeric interactions exist not only between the TM domains or STAS domains but also between the cytoplasmic surfaces of the TM domain and the STAS domain of the opposite chain (Fig. 1 *b*). The determination of the structure of human SLC26A9 was performed using the full-length (FL) protein, although the long intervening loop in the STAS domain is disordered. The overall structure of human SLC26A9 is almost the same as that of mouse SLC26A9. Interestingly, the electron densities of the C-terminal regions are observed at the intracellular vestibule of the putative ion transport pathway, and the C-terminal helices are bound near the entrance of the pathway (Fig. 1 *a*). The electrophysiological analysis showed that the existence of C-terminal helices inhibits the transport of chloride ions and the deletion of the C-terminal helices enhances the transport of chloride ions. In addition, molecular dynamics (MD) simulations demonstrated that chloride ions approach the binding site through the pore region between the core and gate domains (34).

The domain architecture of SLC26A9 has been suggested to be consistent with an elevator motion, a type of alternate-access mechanism, which is the structural rearrangement of transporter proteins to facilitate the movement of the substrate across the membrane (34). Though the flexibility of the TM helices that interact with the substrate has been implicated in transport movement (35), the series of movements before the elevator-like transport pathway, which can be a key feature of the transport mechanism of SLC26A9, is unclear. In addition, mutations in the STAS domain reduce the transport function by affecting activity around the interface between the TM regions (36,37,38,39), but little is known about the role of the STAS domain in chloride ion transport.

In order to better understand the dynamic properties of SLC26A9 and its role in chloride ion transport, we performed MD simulations of FL, STAS domain removal (ΔSTAS), and C-terminus removal (ΔC) models of the human SLC26A9 protein. By analyzing the simulation data, we aimed to investigate the interactions between the STAS domain and the TM region, as well as the effect of the C-terminal helices on chloride ion transport. Our simulations allowed us to observe the stable binding of chloride ions consistent with the putative ion transport pathway. Moreover, our results revealed large and asymmetric motions of the STAS domain that promote conformational changes in the TM domain, facilitating the alternate-access mechanism. The insights gained from our study have important implications for the rational design of new drugs and more effective therapeutic treatments for disorders related to chloride ion transport, such as CF.

## Materials and methods

### Model building

To investigate the effect of the STAS domain and C-terminal helix on chloride ion permeation in SLC26A9, we constructed three models of the protein: FL, ΔSTAS, and ΔC (Fig. 1). We obtained the cryo-EM structure of human SLC26A9 from the PDB (40) (PDB: 7CH1). However, since the intrinsically disordered region of the long intervening loop (residues 568–652) in the STAS domain is disordered in the cryo-EM structure of human SLC26A9, we modeled the truncated portion by replacing it with a loop consisting of residues 568–573 and 648–652 strung together. Additionally, we modeled the short disordered regions, which include residues 1–4, 27–44, 151–157, 742–772, and 785–791, to construct the FL model of residues 1–791. Modeling of all disordered regions was attempted using both the CHARMM GUI (41) and MODELLER v.9.25 (42), and the models without unnatural elongation structures were adopted. Thus, we used CHARMM GUI to model all disordered regions except residues 151–157, which we modeled using MODELLER. All modeled disordered regions adopted had no characteristic secondary structures and were located in solvent-exposed locations. We built the ΔC model by excluding the C-terminal loop and residues 742–791, which include the loop region before and after the C-terminal loop. The ΔSTAS model was created from the ΔC model by excluding residues 1–44 and 500–741 that correspond to the STAS domain.

### MD simulations

We inserted each SLC26A9 model (FL, ΔSTAS, and ΔC) into a 1-palmitoyl-2-oleoyl-sn-glycero-3-phosphocholine model lipid membrane and immersed them in rectangular boxes filled with TIP3P (43) water molecules together with sodium and chloride ions, resulting in a salt concentration of 150 mM. The resulting systems contained 276,960, 209,084, and 277,656 atoms for the FL, ΔSTAS, and ΔC models, respectively. The number of 1-palmitoyl-2-oleoyl-sn-glycero-3-phosphocholine molecules in the upper and lower leaves are 232 and 216, 232 and 224, and 232 and 223 for the FL, ΔSTAS, and ΔC models, respectively. We built all systems using the Membrane builder (44) in CHARMM-GUI (41,45). We performed simulations using the MD program GROMACS v.2018.2 (46). We employed the CHARMM36m force field (47) for proteins and lipids and calculated the long-range electrostatic interactions using the particle-mesh Ewald method (48). We set the switching length for electrostatics and the cutoff length for the Lennard-Jones potential at 10 Å. We treated water molecules and CHx, NHx (x = 1, 2, or 3), SH, and OH groups as rigid bodies using the LINCS (49) to allow a time step of 2.0 fs. We energy minimized each simulation system using a 5000-step steepest descent method, followed by gradually decreasing the restraints on protein and lipid heavy atoms for three consecutive 25 ps NVT simulations. In these simulations, we used a 1 fs time step and maintained a temperature of 303.15 K using a Berendsen temperature-coupling scheme. We then performed three consecutive 100 ps NPT simulations with further gradual release of heavy-atom restraints. In the last 100 ps simulation, the lipids were completely unrestrained and allowed to relax around the restrained protein. In these simulations, we used a 2 fs time step and kept the pressure constant at one bar using a semi-isotropic Berendsen pressure barostat. We performed independent 1 *μ*s production runs twice for FL and ΔSTAS and five times for ΔC.

### Ion-binding probability

The probability of the presence of chloride ions per space was estimated for the entire space of the system as the probability of a chloride ion being located in a 1╳1╳1 Å^3^ grid cell during the 1 *μ*s simulation. The putative ion-binding site was defined as the space within an 8.5 Å radius, 5 times the van der Waals radius of a carbon atom, from the midpoint of the Cα atoms of Phe128 and Leu391, respectively, with reference to the cryo-EM structures of human SLC26A5 in the chloride-binding state (30). Ion binding in the 3D probability distribution map was defined as the presence of a grid cell with a probability of chloride ion presence greater than *μ*+50σ at the putative ion-binding site, where *μ* and σ are the average and the standard deviation of the probability of ion presence in the entire space of the system. Time variation of the probability of chloride ion binding to the binding site was calculated by counting the number of chloride ions that are within 9 Å of the Cα atoms of both Phe128 and Leu391 for each frame.

### Free energy calculation

The free energy profile where the objective event occurs (e.g., ion binding) for the arbitrary reaction coordinate was estimated as follows:

(Equation 1) $$\Delta G_{i}=-RT\;\log\frac{n_{i}}{n_{\min}}\text{,}$$

where *R* is the gas constant and *T* is the temperature. *n*_*i*_ represents the number of times the objective event occurred when the system was in the *i*-th grid cell in the reaction coordinate space, and *n*_min_ represent the smallest non-zero number of times the objective event occurred in all grid cells in the reaction coordinate space. The grid cell interval was set so that *n*_min_ is 1.

## Results and discussion

### Role of STAS domain in ion transport

**Figure 2:**
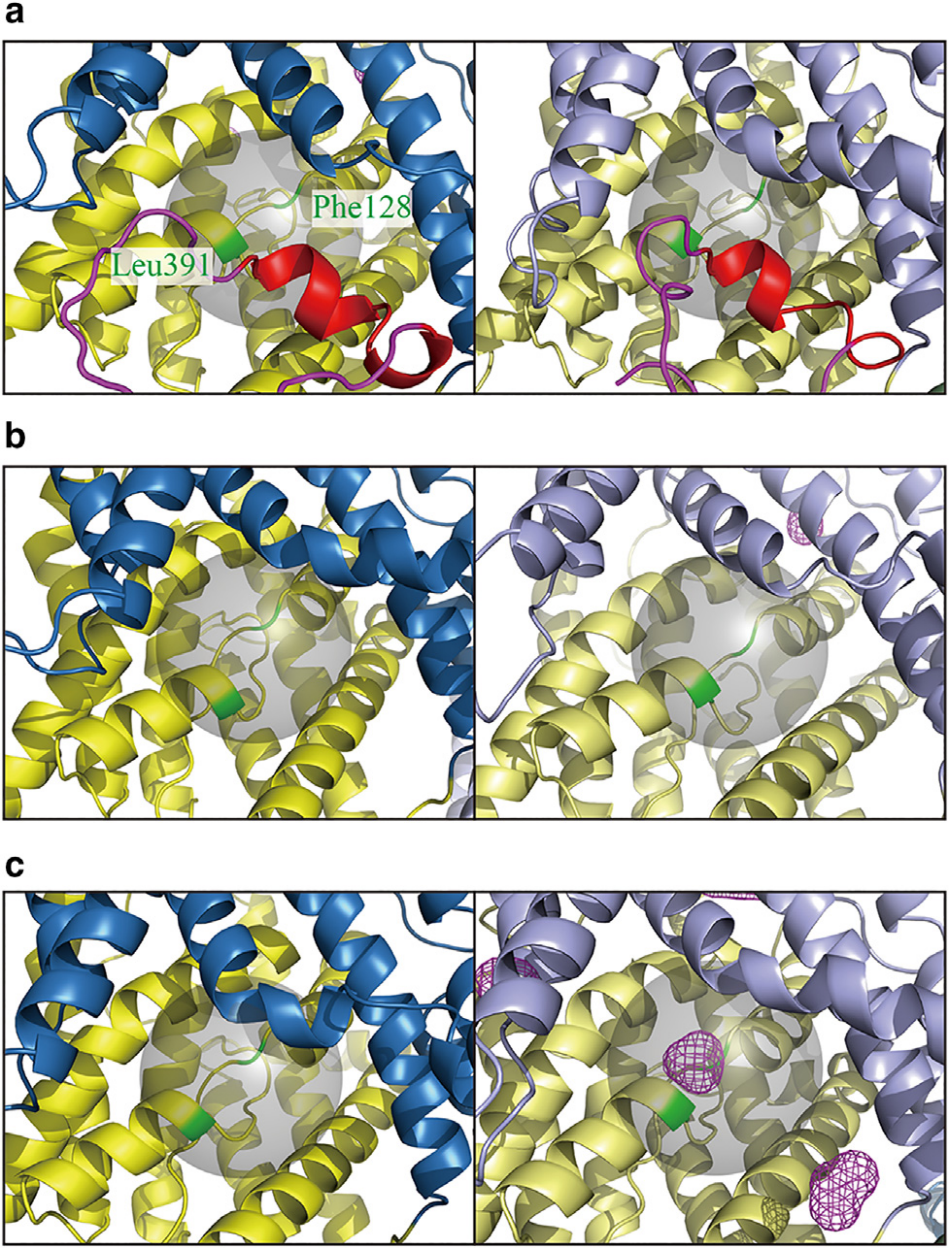
3D probability distribution maps of the chloride ion presence. The cartoon represents the average structure of each trajectory. The colors of the cartoon represent the same elements as in Fig. 1 with the exception of Phe128 and Leu391 of the chloride-ion-binding site, which is colored green. The putative chloride-ion-binding sites are represented by the transparent gray spheres. The spaces with probabilities of chloride ion presence greater than *μ*+50σ are indicated by the magenta meshes. The maps for chain A and chain B are shown on the left and right for each trajectory, respectively. (*a*) FL trajectory 1. (*b*) ΔSTAS trajectory 2. (*c*) ΔC trajectory 1. The probability distribution maps for other trajectories are shown in Fig. S1. To see this figure in color, go online.

In this study, we performed nine independent 1 *μ*s simulations of SLC26A9, including FL × 2, ΔSTAS × 2, and ΔC × 5, in the presence of 150 mM NaCl under NPT ensemble conditions. We analyzed the probabilities of chloride ion present in the putative chloride-ion-binding site for each simulation. 3D probability distribution maps of the chloride ion presence are shown in Figs. 2 and S1, and Table 1 shows whether the ions were bound to the putative ion-binding sites, for each chain in the simulations in this study. It is observed that in the FL (Figs. 2 *a* and S1 *a*) and ΔSTAS (Figs. 2 *b* and S1 *b*) simulations, no significant peaks in the probability of chloride ion presence in the putative chloride-ion-binding sites were identified.

**Table 1:** The presence of the chloride and sodium ion bindings to the putative ion-binding sites and the dual-mode dihedral angular correlations of TM12

Trajectory		Chloride ion binding	Sodium ion binding	Dual-mode dihedral angular correlation
FL1	chain A	–	–	–
	chain B	–	–	–
FL2	chain A	–	–	✓
	chain B	–	–	–
ΔSTAS1	chain A	–	–	✓
	chain B	–	–	✓
ΔSTAS2	chain A	–	–	–
	chain B	–	–	–
ΔC1	chain A	–	–	–
	chain B	✓	–	✓
ΔC2	chain A	–	–	–
	chain B	✓	–	✓
ΔC3	chain A	–	–	✓
	chain B	✓	–	✓
ΔC4	chain A	–	–	–
	chain B	–	–	✓
ΔC5	chain A	–	–	✓
	chain B	✓	–	✓

However, in ΔC simulations, a significant peak in the probability of the presence of chloride ions in the putative chloride-ion-binding sites was observed in four out of the five simulations (ΔC t-1, -2, -3, and -5; Figs. 2 *c* and S1, *c1*–*c4*). As the number of samples increases, the probability of the presence of ions per space will be Poisson distributed, and the expected values of *μ* and σ will be equal. Thus, a probability of ionic presence of *μ*+50σ as shown in Fig. 2 corresponds roughly to an ion concentration of 7.65M (=0.15 ╳ 51M). On the other hand, no significant high probability of sodium ion presence in the putative chloride-ion-binding sites was observed (Fig. S2; Table 1) for all trajectories for the FL-t1-2, ΔSTAS-t1-2, and ΔC-t1-5 simulations. This suggests that the ion-binding site of SLC26A9 specifically binds only chloride ions. The chloride ion was found to be interacting with the main chains of THR127, PHE128, ALA390, LEU391, and SER392 and side chains of GLN88, PHE128, ALA390, LEU391, SER392, ASN441, and ASN444, which is consistent with the cryo-EM structures of human SLC26A5 in the chloride-binding state (30) (Fig. S3 *a*).

The peak location of the probability of the presence of chloride ions is presumed to be the chloride-ion-binding site of SLC26A9. From the mutational analysis, it was revealed that the residues (GLN88, PHE92, THR127, PHE128, LEU391, SER392) surrounding the chloride ion have a significant impact on the transport characteristics of SLC26A9 (33). Importantly, the probability of the presence of chloride ions is almost zero at a position slightly closer to the extracellular side of the TM domain than the binding site, indicating that the chloride ions entered the binding site from the cytoplasmic side and that these SLC26A9 structures are inward-facing open structures.

To transport chloride ions from the cytoplasmic side of the plasma membrane to the extracellular side in an alternate-access mechanism, chloride ions must be stably bound inside SLC26A9 for a long time while SLC26A9 changes from an inward open structure to an outward open structure. For trajectory 1 of ΔC, where the peak in the probability of chloride ion presence was observed to be most pronounced, the time variation of the probability of chloride ion binding to the binding site shown in Fig. S3 *b* and Video S1 indicates that stable ion binding has occurred over a long period of time. Therefore, the ΔC simulation demonstrated stable binding of chloride ions, which is necessary for TM transport of ions, suggesting that the alternate-access mechanism operates.

Video S1. Binding of a chloride ion to SLC26A9

In summary, our simulations indicate that stable binding of chloride ions was observed only in ΔC, which may reflect the alternate-access mechanism. The C-terminal helix in the FL model plugged the ion pathway and inhibited chloride ions from accessing the binding site. Furthermore, the absence of stable binding of chloride ions in the ΔSTAS model suggests that the STAS domain is required for chloride ion transport. These observations are consistent with findings in previous studies (34,36,37,38,39,50). In the following studies, we further analyze the role of the STAS domain in regulating the access of chloride ions to the binding sites.

### Asymmetric motion of STAS domain promotes the gating of SLC26A9

Our simulations revealed that the TM domains of the SLC26A9 dimer exhibit asymmetric behavior in the absence of the C-terminal helix, suggesting that the STAS domain plays a crucial role in maintaining the distance between the core domain and the gate domain. Figs. 2 and S1 show that the chloride ions bound to binding sites in four of the five ΔC model simulations. However, in all those simulations, the chloride ion only accessed the binding site on one of the SLC26A9 dimers, suggesting that the gating mechanism may be asymmetric.

**Figure 3:**
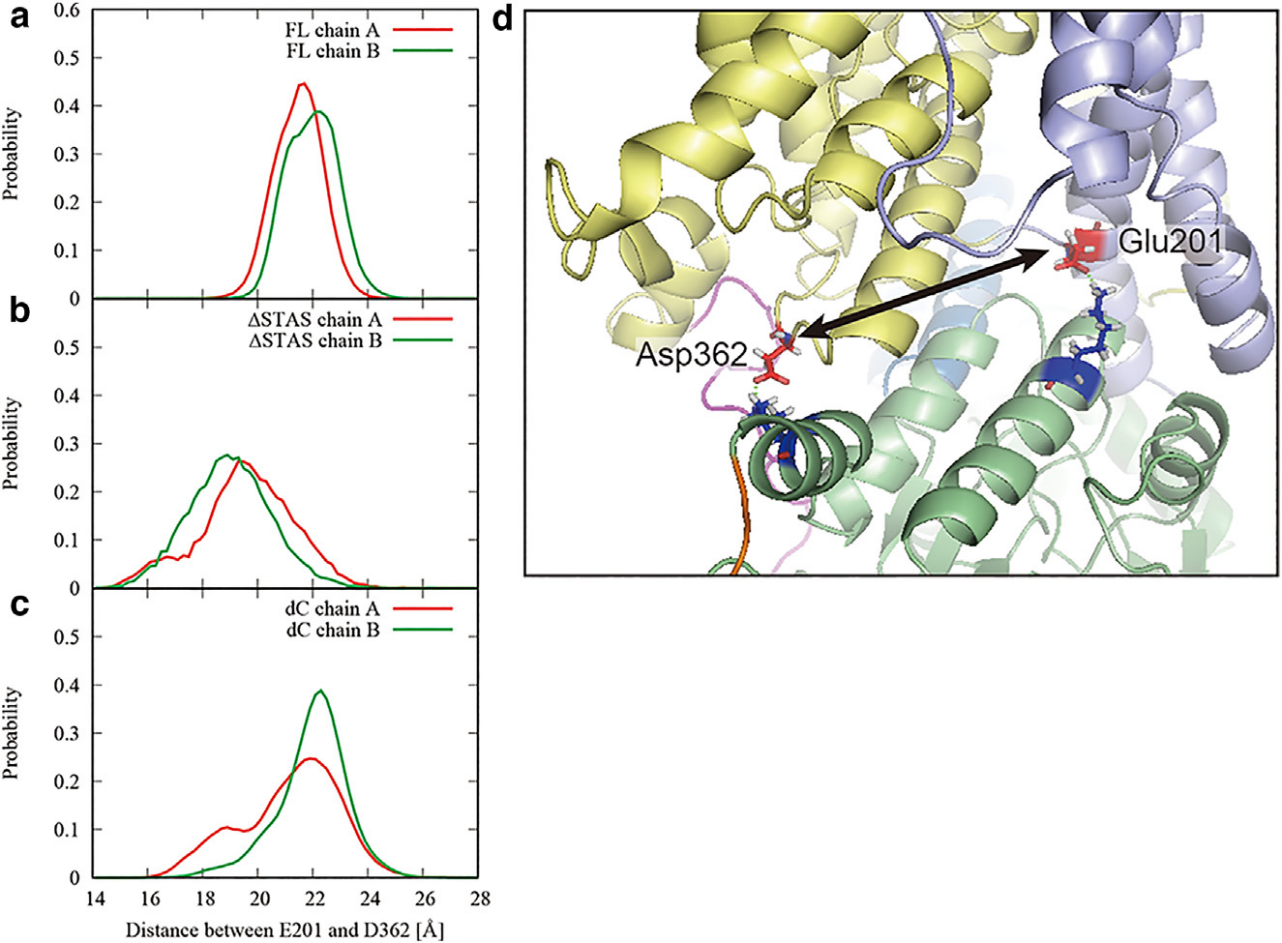
Distance between the core domain and the gate domain. Distributions of the distance between the core domain and the gate domain for chains A (*red*) and B (*green*) were calculated using all trajectories for the (*a*) FL-t1-2, (*b*) ΔSTAS-t1-2, and (*c*) ΔC-t1-5 simulations. (*d*) Distance between the core domain and the gate domain was defined as the distance between the Cα atoms of ASP362 and GLU201. The colors of the cartoon represent the same elements as in Fig. 1. Stick representation of basic (*blue*) and acidic (*red*) residues are also shown. Salt bridges are indicated by the green dotted lines. To see this figure in color, go online.

Fig. 3, *a*–*c*, present the distribution of the distance between the core domain and the gate domain for the chains A and B using all trajectories for the FL-t1-2, ΔSTAS-t1-2, and ΔC-t1-5 simulations, respectively. The distance between the core domain and the gate domain was calculated as the distance between the Cα atoms of ASP362 and GLU201 located at the respective tips of the core and gate domains (Fig. 3 *d*). The distance in the initial structure was about 22 Å. In the FL simulations, the distances between the core domain and the gate domain did not change significantly from the initial structure for both the chains A and B (Fig. 3 *a*). On the other hand, in the ΔSTAS simulations, although the structure of the TM domains of individual chains and the approximate positional relationship of the dimer were maintained (Fig. S4, *b1* and *b2*), the distances between the core domain and the gate domain for both chains A and B were smaller than in the initial structure (Fig. 3 *b*), indicating that the STAS domain helps to maintain the distance between the core and the gate domains.

Furthermore, the ΔC simulation showed asymmetric behavior, with chain A having a smaller distance between the core and gate domains than the initial structure, while chain B had a slightly larger distance than the initial structure (Fig. 3 *c*). This observation implies that not only the STAS domain but also the C-terminal helix affect the dynamic behavior of the TM domain of SLC26A9. This difference in behavior may be attributed to the fact that the C-terminal helix plays a role in stabilizing the STAS domain and restricting its motion, as we will discuss next.

**Figure 4:**
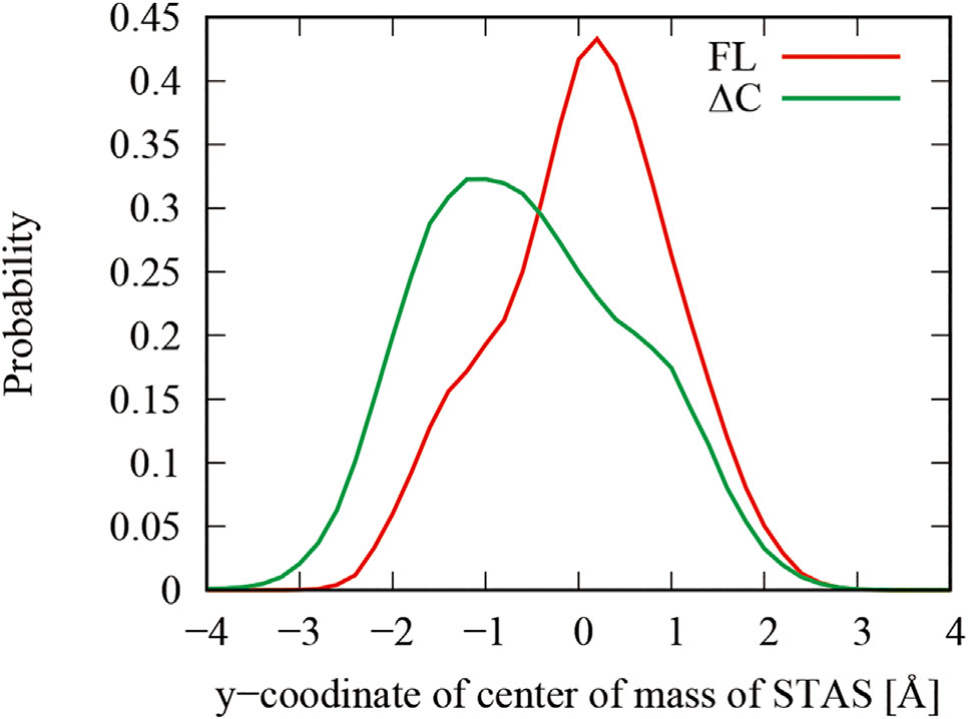
Positional distributions of the center of mass of the STAS domain calculate from all trajectories for the FL-t1-2 (*red*) and ΔC-t1-5 (*green*). The histogram represents the *y* coordinates of the positions of the center of mass of the STAS domains when the line connecting the respective centers of mass of the transmembrane domains of A and B chains is the *x* axis. To see this figure in color, go online.

To better understand the role of the STAS domain in promoting the gating of SLC26A9, we analyzed the positional distributions of the center of mass of the STAS domain along the *y* axis. This axis is defined as the outer product of the *z* axis and the *x* axis, where the *z* axis is perpendicular to the membrane, running from the cytoplasm to the extracellular space, and the *x* axis is determined by the component perpendicular to the *z* axis of the vector originating from the center of mass of the TM domain of chain B to that of chain A. This analysis was conducted for the all trajectories for the FL-t1-2 and ΔC-t1-5 simulations (Fig. 4). Root-mean-square deviation profiles of the TM and STAS domains for all simulations are shown in Fig. S4.

**Figure 5:**
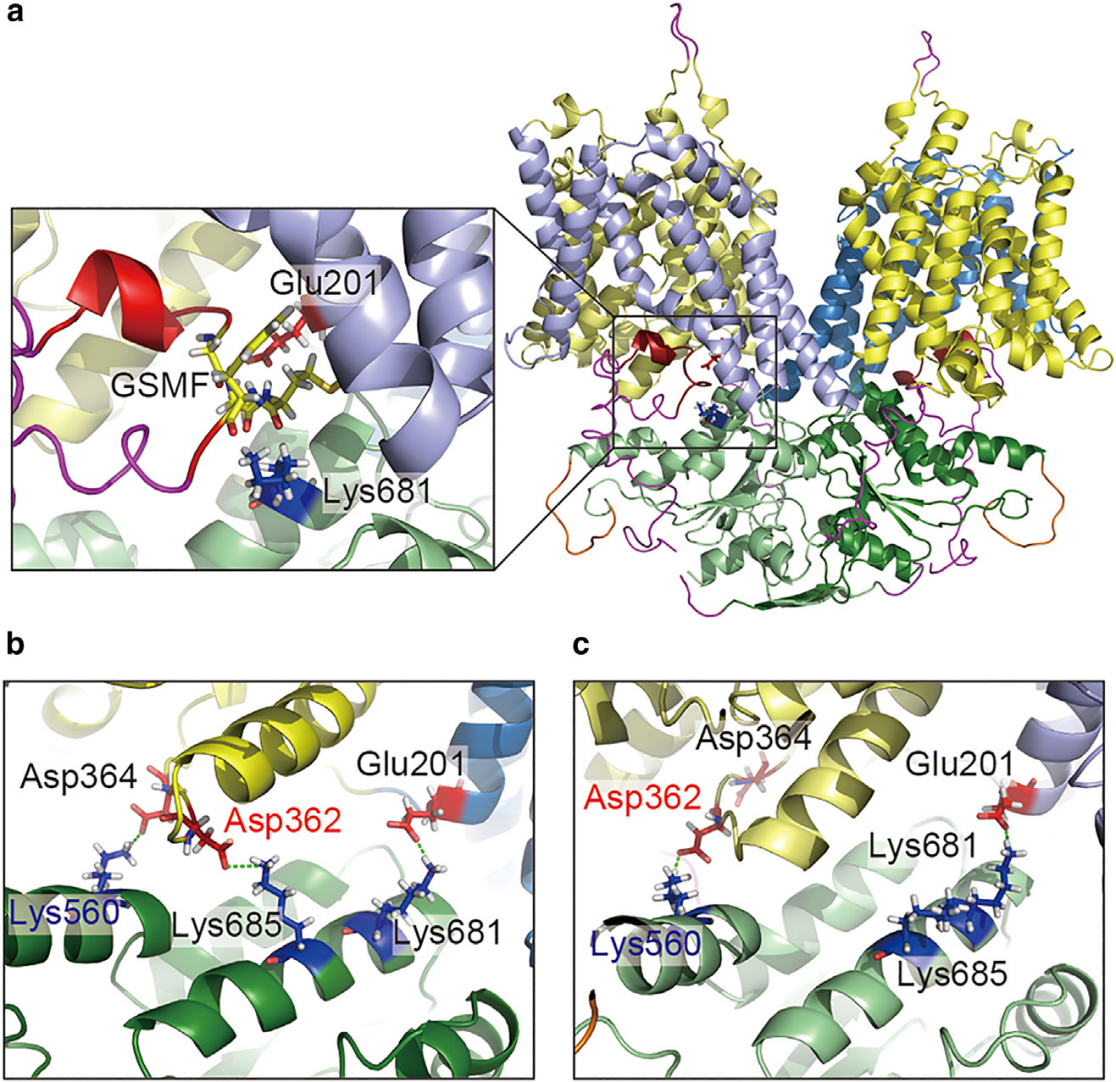
Different salt bridge formations between chain A and chain B resulting from the deletion of the C-terminus. (*a*) Close-up view around the C-terminal helix of the SLC26A FL model. (*b* and *c*) Snapshots from (*b*) chain A side and (*c*) chain B side of ΔC-t1 simulation at 664 ns. The colors of the cartoon represent the same elements as in Fig. 1. Stick representation of basic (*blue*), acidic (*red*), and PDZ binding motif: GSMF (*yellow*) residues are also shown. Salt bridges are indicated by the green dotted lines. To see this figure in color, go online.

We found that the STAS domain of ΔC has a larger (Fig. S4, *a1*, *a2*, and *c1*–*c5*) and more asymmetric motion than the STAS domain of FL (Fig. 4). In FL, the PDZ-binding motif in the C-terminal region is stuck between the GLU201 in the gate domain and LYS681 in the STAS domain (Fig. 5 *a*), thus restricting the movement of the STAS domain. In contrast, in the case of ΔC, the deletion of the C-terminal helix is thought to create a salt bridge between GLU201 and LYS681, which may excite the motion of the STAS domain. This large and asymmetric motion of the STAS domain of ΔC resulted in different salt bridge formations between the chains, as we discuss next.

**Figure 6:**
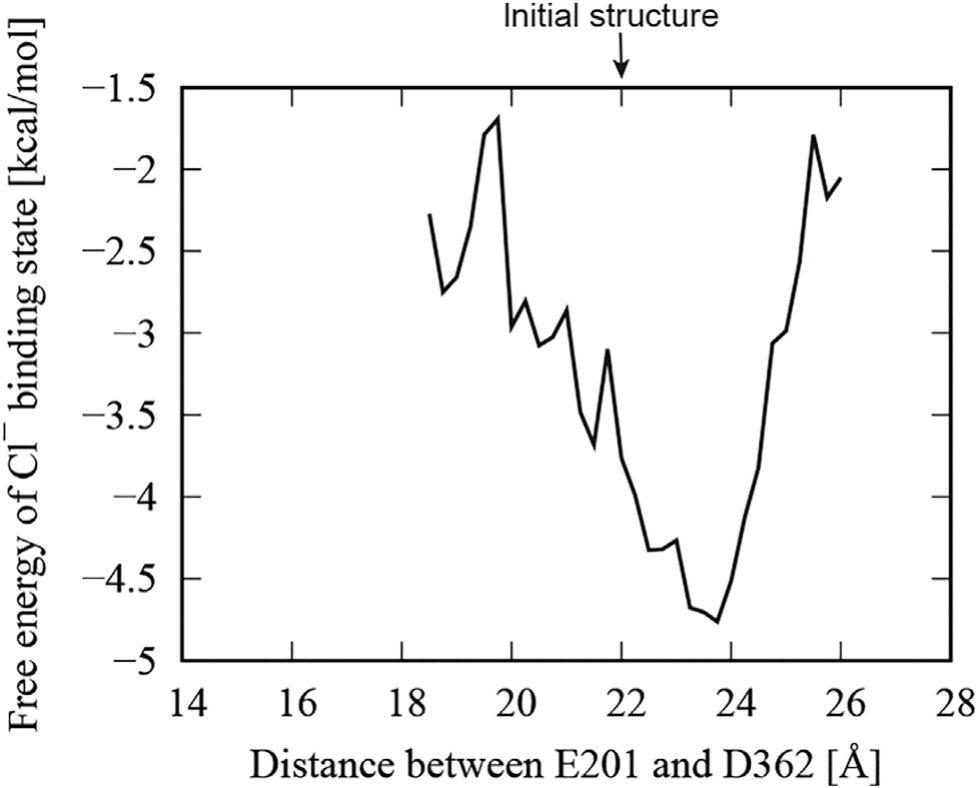
Free energy landscape of chloride ion binding versus the distance between the core and gate domains calculated from five ΔC simulations.

In the ΔC-t1 simulation, in the TM domain of chain A, ASP362 in the core domain of chain A formed a salt bridge with LYS685 in the STAS domain of chain B, and ASP364 in the core domain of chain A formed a salt bridge with LYS560 in the STAS domain of chain B (Fig. 5 *b*), whereas in the TM domain of chain B, ASP362 in the core domain of chain B and LYS560 in the STAS domain of chain A formed a salt bridge (Fig. 5 *c*). This difference in salt bridge formation may have resulted in the difference in the relative positions of the core and gate domains between chains A and B. Fig. 6 shows the free energy landscape of chloride ion binding versus the distance between the core and gate domains, calculated from five ΔC simulations. The free energy was calculated by Eq. 1 with the distance between the core and the gate domains as the reaction coordinate and the binding of chloride ions as the objective event. The grid cell interval in the reaction coordinate space was set to 0.25 Å. This free energy landscape means that the binding of chloride ions favors a structure in which the distance between the core and gate domains is slightly larger than in the initial structure. The increase in the distance between the core domain and the gate domain corresponds to the gating of the pathway through which chloride ions are transported.

To summarize, our findings suggest that the removal of the C-terminal helix triggers asymmetric motion of the STAS domain, which ultimately opens the gate of the TM domain and enables chloride ions to bind to the binding site. The small motion of the STAS domain in ΔC-t4, the only ΔC simulation in which no binding of chloride ions to the binding site was observed, also suggests that the motion of the STAS domain is essential for the promotion of ion binding (Fig. S4 *c*4). However, it remains unclear how ΔC stably binds chloride ions to the binding site. In the following section, we analyze how the STAS domain participates in the ion transport by the alternating-access mechanism.

### Stretching of the TM12 helix stabilizes the binding of chloride ion

**Figure 7:**
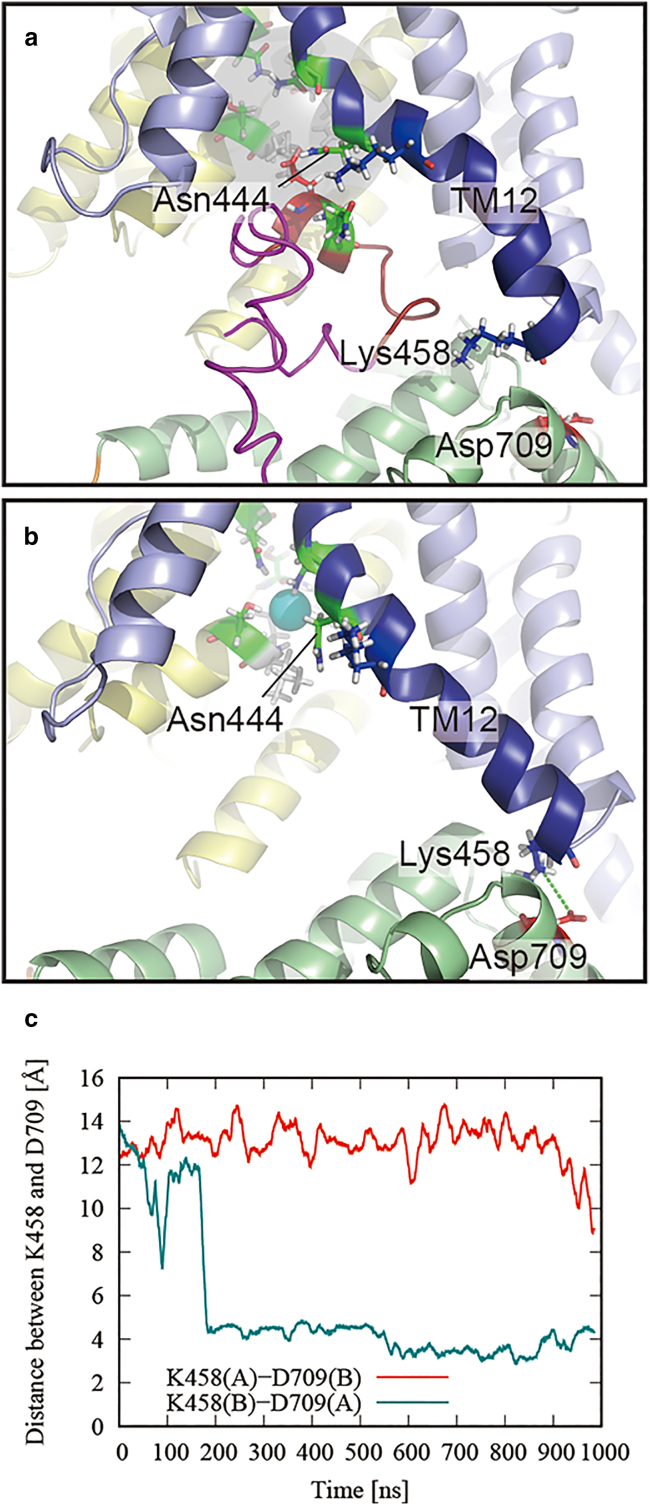
Stretching of the TM12 helix by the deletion of the C-terminus. (*a*) Close-up view around the C-terminal helix of the SLC26A9 cryo-EM structure (PDB: 7CH1) and (*b*) snapshot of the same view at 750 ns. The colors of the cartoon represent the same elements as in Fig. 1 with the exception of the TM12 helix of the gate domain, which is colored deep blue. The C-terminal ordered and disordered regions are shown in dark red and magenta, respectively. Stick representations of basic (*blue*), acidic (*red*), polar (*green*), and hydrophobic (*white*) residues are also shown. Putative ion-binding site and bound chloride ion are represented by gray transparent sphere and cyan van der Waals sphere, respectively. Salt bridges are indicated by the green dotted lines. (*c*) Time variation of distance between the Nζ atom of LYS458 at the end of the TM12 helix of chain B and the Oδ atom of ASP709 at the interface of the STAS domain of chain A in the ΔC-t1 simulation. To see this figure in color, go online.

In this section, we discuss how the stretching of the TM12 helix stabilizes the binding of chloride ion in the ΔC simulation. The TM12 helix is a gate domain helix that interacts with the C-terminal helix to form a kinked structure in the cryo-EM structure. In the initial structure, LYS447 and GLU775 interacted by a salt bridge (Fig. 7 *a*). In the ΔC simulation, the asymmetric motion of the STAS domain leads to the formation of a salt bridge between the TM12 and STAS domains (Fig. 7 *b*). This salt bridge formation is also observed in the time variation of the distance between the Nζ atom of LYS458 at the end of the TM12 helix of chain B and the Oδ atom of ASP709 at the interface of the STAS domain of chain A in the ΔC-t1 simulation (Fig. 7 *c*).

**Figure 8:**
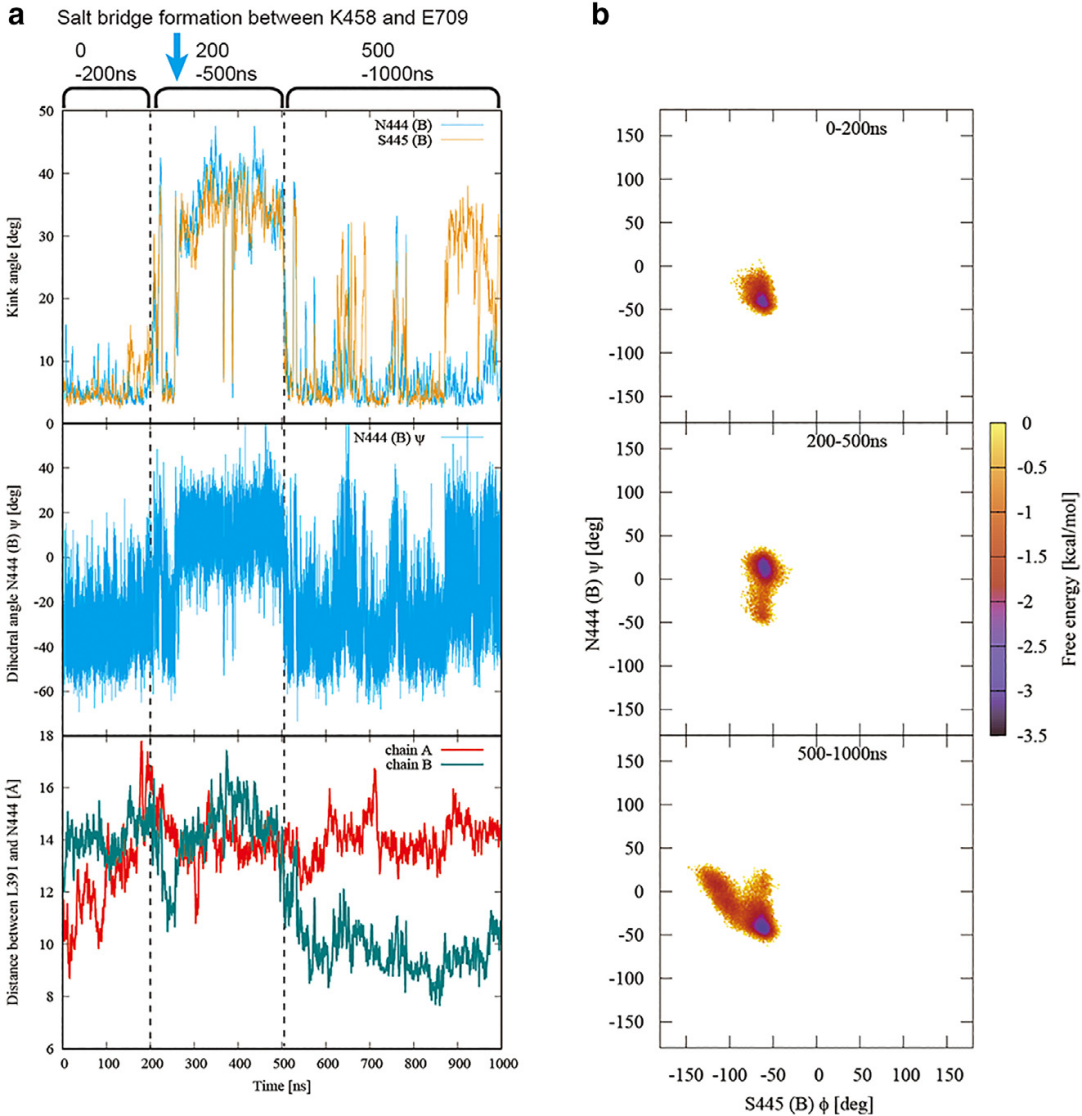
Stiffening of the TM12 helix to stabilize the ion binding. (*a*) Quantitative values for the deformation of the TM12 helix in the ΔC-t1 simulation. The time point when the salt bridge is formed between LYS458 (chain B) and GLU709 (chain A) is indicated by the cyan arrow. The time point when large changes in kink angles occur is indicated by the dashed line. (*Top*) Time variation of kink angle for ASN444 (chain B) (*cyan*) and SER445 (chain B) (*orange*). (*Middle*) Time variation of main chain dihedral angle ASN444ψ (chain B). (*Bottom*) Time variation of the distance between the Cα atoms of LEU391 and ASN444 in chains A (*red*) and B (*green*). (*b*) Free energy landscape showing the correlation between the dihedral angles SER445φ and ASN444ψ of chain B in the ΔC-t1 simulation. (*Top*) 0–200 ns. (*Middle*) 200–500 ns. (*Bottom*) 500–1000 ns. To see this figure in color, go online.

As shown in Figs. 4 and S4, ΔC has a larger STAS motion than that of FL. In addition, as shown in Fig. 7, *a* and *b*, removing the C-terminus from between the core and gate domains creates a spatial clearance where the TM12 helix can move freely. These increases in the mobility of the STAS domain and the TM12 helix would increase the probability of a stochastic transition in which a salt bridge is formed between the STAS domain and the TM12 helix, as shown in Fig. 7. The TM12 helix is kinked in its initial structure, but this salt bridge would increase the probability of having a stretched structure as shown in Fig. 2 *b*. Fig. 8 *a* quantifies the stretching of the TM12 helix in the ΔC-t1 simulation. The helix kink is calculated as the angle between the helix axis segments. ASN444 and SER445 in the TM12 helix of chain B were not kinked in the initial structure but kinked significantly at 200 ns, coinciding with the formation of a salt bridge between LYS458 (B) and GLU709 (A) (Fig. 8 *a*, *top*). At the same time, the main-chain dihedral angle ASN444ψ (B) also changed significantly (Fig. 8 *a*, *middle*). However, at this point in chain B, there is no significant change in the distance between LEU128, which is in the core domain and forms a chloride-ion-binding site, and ASN444 of the TM12 helix (Fig. 8 *a*, *bottom*). Therefore, the change in kink and dihedral angles of the TM12 helix at 200 ns is not considered to be involved in stabilizing chloride ion binding. Subsequently, under the influence of STAS motion, the kink angles of ASN444 and SER445 in chain B become smaller again at 500 ns (Fig. 8 *a*, *top*), and at the same time, ASN444ψ changes significantly (Fig. 8 *a*, *middle*). From this 500 ns point, the distance between LEU391 and ASN444 in chain B becomes smaller (Fig. 8 *a*, *bottom*).

The smaller distance between LEU391 and ASN444 facilitates the formation of clusters of hydrophilic amino acids around the chloride-ion-binding sites of the core domain, as shown in Fig. S3 *a*. This configuration allows for the stable binding of chloride ions, as depicted in Fig. S3 *b*. Therefore, the stretching of the TM12 helix at 500 ns, accompanied by changes in kink angle and dihedral angle, played a significant role in stabilizing the binding of chloride ions. These results indicate that the TM12 helix exhibited distinct properties at 200 and 500 ns. Fig. 8 *b* presents a free energy landscape that reveals the correlation between the dihedral angles SER445φ and ASN444ψ in the ΔC-t1 simulation. The free energy was calculated by Eq. 1 with SER445φ and ASN444ψ as the 2D reaction coordinate and the event for which the combination of SER445φ and ASN444ψ is within the range of the respective grid cell in the reaction coordinate space as the objective event. The grid cell interval in the reaction coordinate space was set to 1°. The changes in both SER445φ and ASN444ψ during the 0–200 ns interval were small, suggesting that the structure of the TM12 helix remained relatively unchanged (Fig. 8 *b*, *top*). However, after ASN444 and SER445 significantly kinked due to the formation of the salt bridge between LYS458 (chain B) and GLU709 (chain A) at 200 ns (Fig. 8 *a*, *top*), ASN444ψ changed significantly in an uncorrelated manner with SER445φ during 200–500 ns (Fig. 8 *b*, *middle*). The uncorrelated change in dihedral angle implies that the structure of the TM12 helix was flexible during this period, and therefore the cluster of hydrophilic amino acids surrounding the chloride-ion-binding site may not have formed. Conversely, after the kink angle between ASN444 and SER445 became small again at 500 ns (Fig. 8 *a*, *top*), ASN444ψ and SER445φ changed in an inversely correlated manner (Fig. 8 *b*, *bottom*). This motion is known as crankshaft motion (51), which maintains the overall structure of the protein despite the large dihedral displacement. In other words, crankshaft motion imparts stiffness to the structure. The TM12 helix exhibits a stiff structure, which is thought to be pulled by the movement of the STAS domain toward the chloride-ion-binding site (Fig. 8 *a*, *bottom*). This, in turn, promotes the formation of the cluster of hydrophilic amino acids surrounding the chloride-ion-binding site, stabilizing the binding of chloride ions (Figs. S3 *a* and 8 *a*, *bottom*).

The dihedral angle motion of the TM12 helix in SLC26A9 depends on the angle between the main chains of adjacent residues. In other words, the TM12 helix switches the dihedral correlation between noncrankshaft and crankshaft modes by changing the shape of the kink. Table 1 shows the relationship between the dihedral angular correlation of TM12 and the presence or absence of stable binding of chloride ions for each chain in the simulations in this study. The correlation of the dihedral angles with both noncrankshaft and crankshaft correlations is denoted as dual-mode correlations. The dihedral angular correlation is defined as a dual-mode correlation when the free energies in SER445φ versus ASN444ψ space are all less than −0.9 kcal/mol at the three points where the combinations of SER445φ and ASN444ψ are {−60°, 15°}, {−60°, −45°}, and {−90°, −15°}. Dual mode correlations typically have a V-shaped profile in the free energy landscape (Fig. S5). The TM12 helix in the chains where chloride ion binding was observed had dual-mode dihedral correlations. This suggests that the dual-mode dihedral angular correlation brought about by the kinked structure of the TM12 helix is required for stable binding of chloride ions.

**Figure 9:**
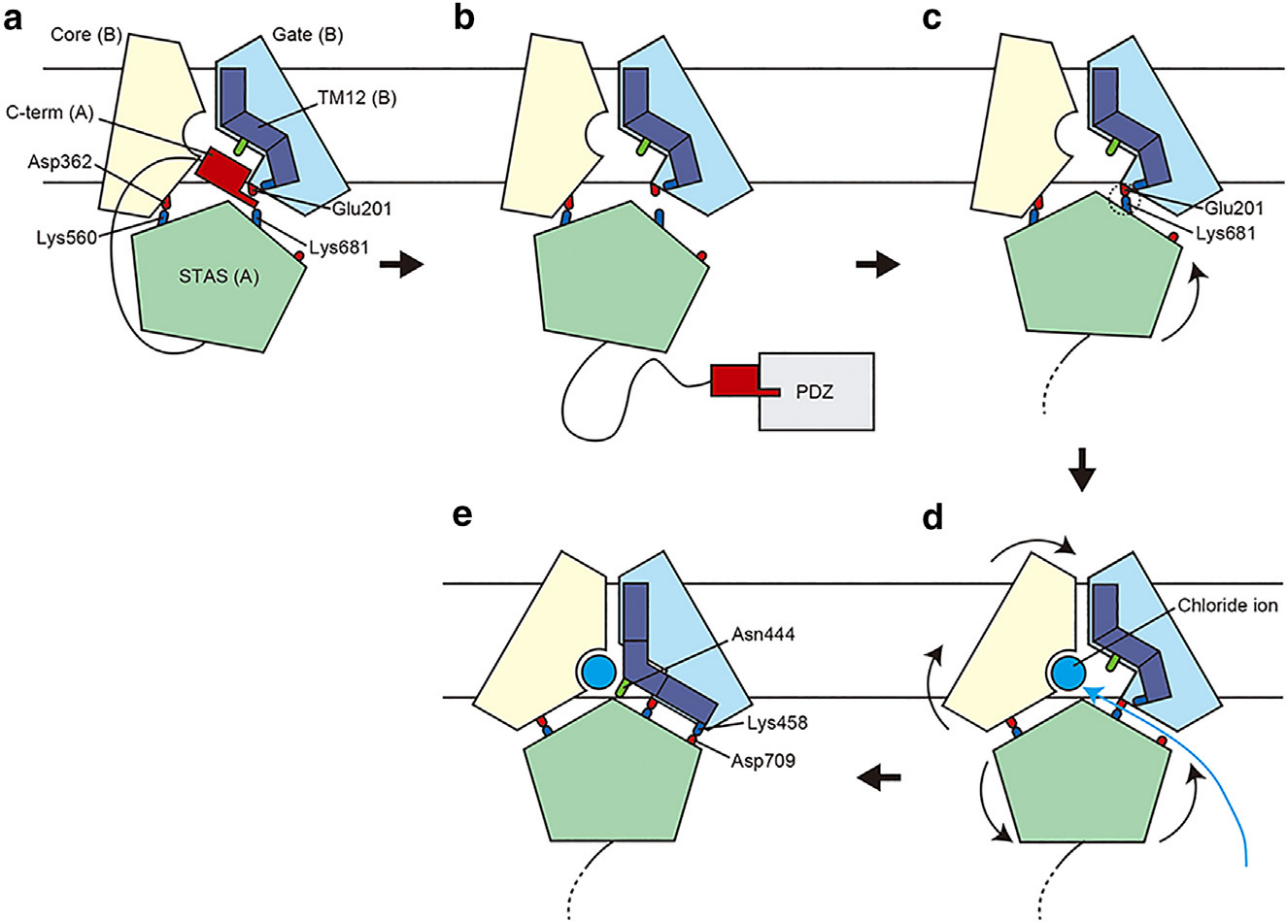
Schematic view of alternate-access mechanism of SLC26A9. The viewing angle is based on that on the left in Fig. 1*a*. Core domain, gate domain, and TM12 helix of chain B are shown in pale yellow, light blue, and deep blue, respectively. The C-terminal helix and the STAS domain of chain A, the PDZ domain of the scaffold protein, and the chloride ion are shown in dark red, light green, gray, and cyan, respectively. Basic, acidic, and polar residues are shown as blue, red, and green sticks, respectively. Salt bridges are represented by dashed circles. Locations of the headgroups of the lipid membranes are indicated as the black solid lines. (*a*) Before insertion of SLC26A9 into the apical plasma membrane. (*b*) C-terminus dissociates from the intracellular vestibule and interacts with the PDZ domain of the scaffold protein after insertion of SLC26A9 into the apical plasma membrane. (*c*) Salt bridge between the gate domain and the STAS domain is created by the removal of the C-terminal helix. (*d*) Distance expansion between core and gate domains caused by asymmetric motion of the STAS domain, allowing chloride ions to enter their binding site. (*e*) Stretching of TM12 by contact with STAS domain forms hydrophilic clusters for stable binding of chloride ions. Such TM12 helix closing motions independent for the core and gate domains are considered essential for alternate-access mechanism. To see this figure in color, go online.

Combining the results so far in this study and knowledge from existing studies, the alternate-access mechanism of SLC26A9 can be interpreted as shown in the schematic view in Fig. 9. Before the insertion of SLC26A9 into the apical plasma membrane, the C-terminal helix is in the chloride ion pathway between the core and gate domains (Fig. 9 *a*). Although the sequence of the C-terminal region in the SLC26 family is not well conserved, some SLC26 transporters harbor a PDZ domain binding motif at their C-termini. SLC26A9 has an X-S/T-X-Φ of class I type PDZ domain binding motif, where Φ represents a hydrophobic residue, and interacts with the PDZ domain of the scaffold protein NHERF1. From existing research, after the insertion of SLC26A9 into the apical plasma membrane, its C-terminus is expected to dissociate from the intracellular vestibule and interact with NHERF1 (Fig. 9 *b*). The salt bridge between the gate domain and the STAS domain is created by the dissociation of the C-terminal helix, thereby triggering the asymmetric motion of the STAS domain (Fig. 9 *c*). The asymmetric motion of the STAS domain causes gating that increases the distance between the core domain and the gate domain, allowing chloride ions to enter their binding site (Fig. 9 *d*). In addition, the TM12 helix interacts with the STAS domain by the salt bridge and transforms the kink structure into a stiff structure and forms hydrophilic clusters for stable binding of chloride ions (Fig. 9 *e*). Independently of the core and gate domains changing to a more inward-open structure than the initial structure, the TM12 helix closes like a lid on the ion binding site to hold chloride ions, suggesting that the observed motion was an initial process of the ion transport by the alternating-access mechanism. If SLC26A9 changes to an outward-open conformation while chloride ions remain bound to the binding site, then it would be able to transport chloride ions from the cytoplasm to the extracellular environment.

## Conclusion

This study sheds light on the alternate-access mechanism in the chloride ion transporter SLC26A9. We found that removing the C-terminal helix not only opens the ion pathway but also initiates STAS domain motion, leading to asymmetric gating of the TM domain and stiffening of the flexible helix near the ion-binding site. This structural change allows stable chloride ion binding, essential for the alternate-access mechanism, and suggests consistency with the initial step of an elevator motion. Our findings lay a robust groundwork for further research into the ion transport mechanisms of SLC family proteins.

In conclusion, we have clarified how the STAS domain and TM12 helix contribute to stabilizing chloride ion binding in the SLC26A9 transporter. The stretching of the TM12 helix and its interaction with the STAS domain form hydrophilic clusters that facilitate stable ion binding. These insights not only enrich our comprehension of SLC26A9’s function but also offer potential guidance for therapeutic strategies addressing diseases related to chloride ion transport dysfunction.

## Data and code availability

The data that support the findings of this study are available from the corresponding author upon reasonable request.

## Author contributions

The contributions of each author to this work are as follows: H.N. conceptualized and proposed the idea for the research study. Y.H. was responsible for selecting the subjects. S.O. performed all calculations and was heavily involved in the data analysis, along with all the other authors. S.O. and Y.H. also took the lead in writing the manuscript. K.K. provided overall supervision, revised the manuscript, ensured coherence and integrity in the study, secured the budget, and managed the project.

## Supporting material

Document S1. Figures S1–S5

Document S2. Article plus supporting material
