# Supplementary material for: The role of the STAS domain in SLC26A9 for chloride ion transporter function
Source: Biophys J. 2024 May 21;123(12):1751–62. doi: 10.1016/j.bpj.2024.05.018 (PMC11214054; doi:10.1016/j.bpj.2024.05.018)
Supplement: Document S1. Figures S1–S5 [file mmc1.pdf]

Biophysical Journal, Volume *123*

Supplemental information

The role of the STAS domain in SLC26A9 for chloride ion transporter function

Satoshi Omori, Yuya Hanazono, Hafumi Nishi, and Kengo Kinoshita

The Role of the STAS domain in SLC26A9 for Chloride Ion Transporter Function

Supplementary Information

Satoshi Omori,[1,2,7] Yuya Hanazono,[1,3,7] Hafumi Nishi,[1,4, 5] Kengo Kinoshita, [1,5,6,*]

Running title: MD simulation of human SLC26A9

[1]Graduate School of Information Sciences, Tohoku University, Sendai, Miyagi 980-8579, Japan.
[2]Department of Bioscience, Nagahama Institute of Bio-Science and Technology, Nagahama, Shiga 526-0829, Japan.
[3]Medical Research Institute, Tokyo Medical and Dental University, Bunkyo-ku, Tokyo 113-8510, Japan.
[4]Faculty of Core Research, Ochanomizu University, Tokyo 112-8610, Japan
[5]Tohoku Medical Megabank Organization, Tohoku University, Sendai, Miyagi 980-8573, Japan.
[6]Institute of Development, Aging, and Cancer, Tohoku University, Sendai, Miyagi 980-8575, Japan.
[7]These authors contributed equally to this work.
*Correspondence: kengo@tohoku.ac.jp

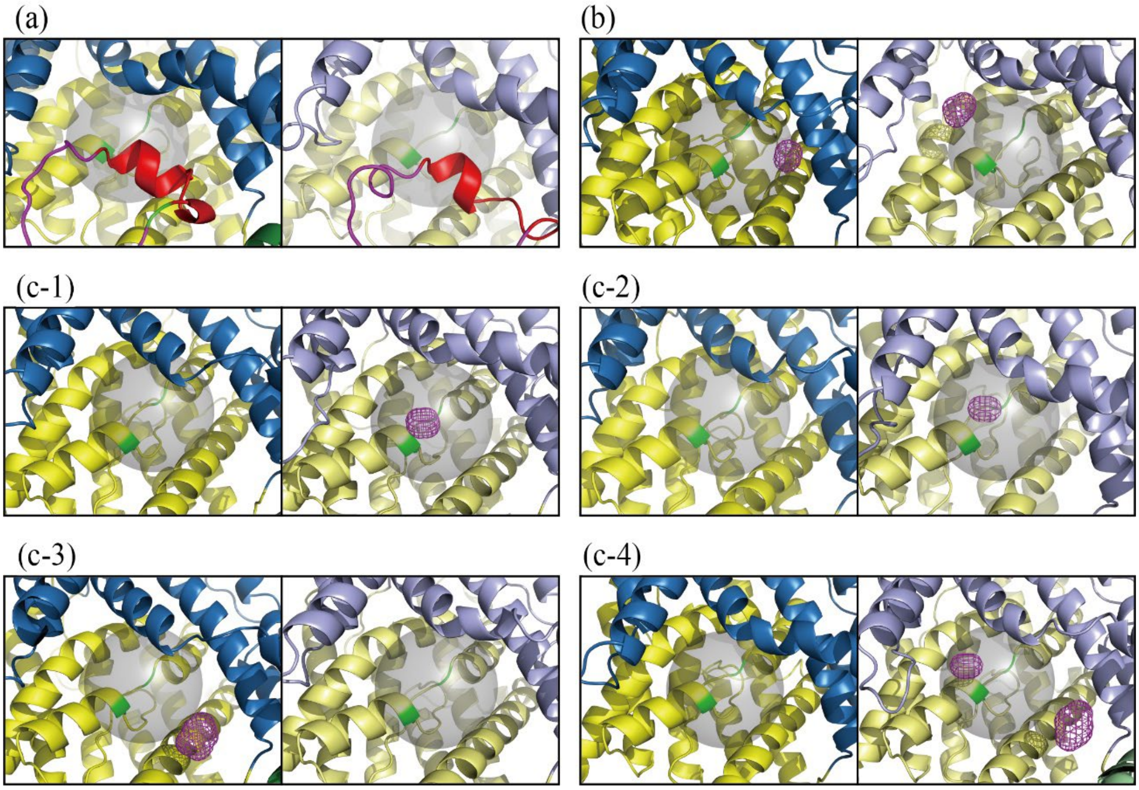

FIGURE S1. 3D probability distribution maps of the chloride ion presence. The cartoon represents the average structure of each trajectory. The colors of the cartoon represent the same elements as in Figure 1, with the exception of Phe128 and Leu391 of the chloride ion binding site which is colored green. The putative chloride ion binding sites are represented by the transparent gray spheres. The spaces with probabilities of chloride ion presence greater than $\mu+50\sigma$ are indicated by the magenta meshes. The map for the chain A and the chain B is shown on the left and right for each trajectory, respectively. (a) FL trajectory 2. (b) ΔSTAS trajectory 1. (c1-4) ΔC trajectory 2-5.

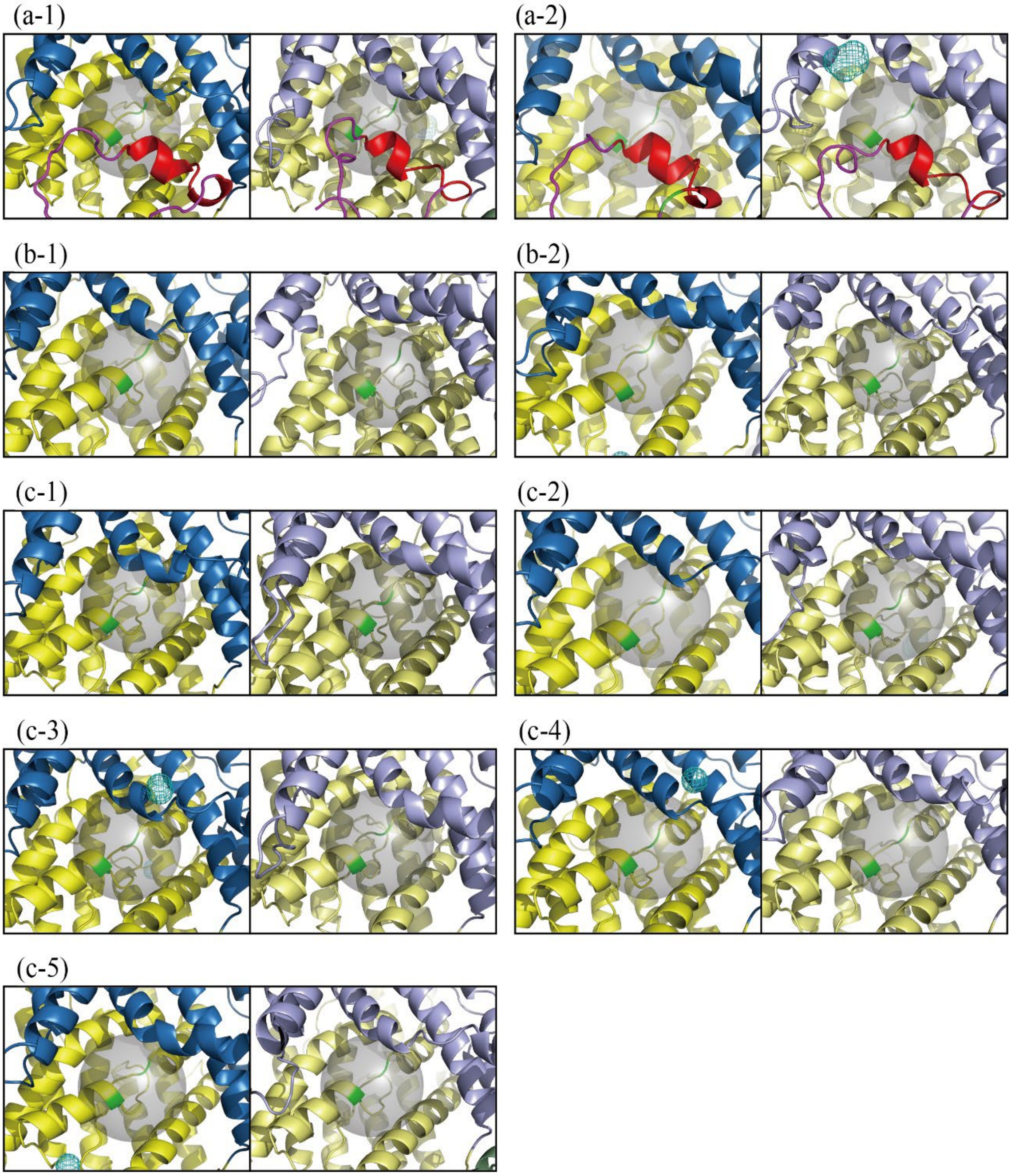

FIGURE S2. 3D probability distribution maps of the sodium ion presence. The cartoon represents the average structure of each trajectory. The colors of the cartoon represent the same elements as in Figure 1, with the exception of Phe128 and Leu391 of the chloride ion binding site which is colored green. The putative chloride ion binding sites are represented by the transparent gray spheres. The spaces with probabilities of sodium ion presence greater than μ+50σ are indicated by the cyan meshes. The map for the chain A and the chain B is shown on the left and right for each trajectory, respectively. (a1-2) FL trajectory 1-2. (b1-2) ΔSTAS trajectory 1-2. (c1-5) ΔC trajectory 1-5.

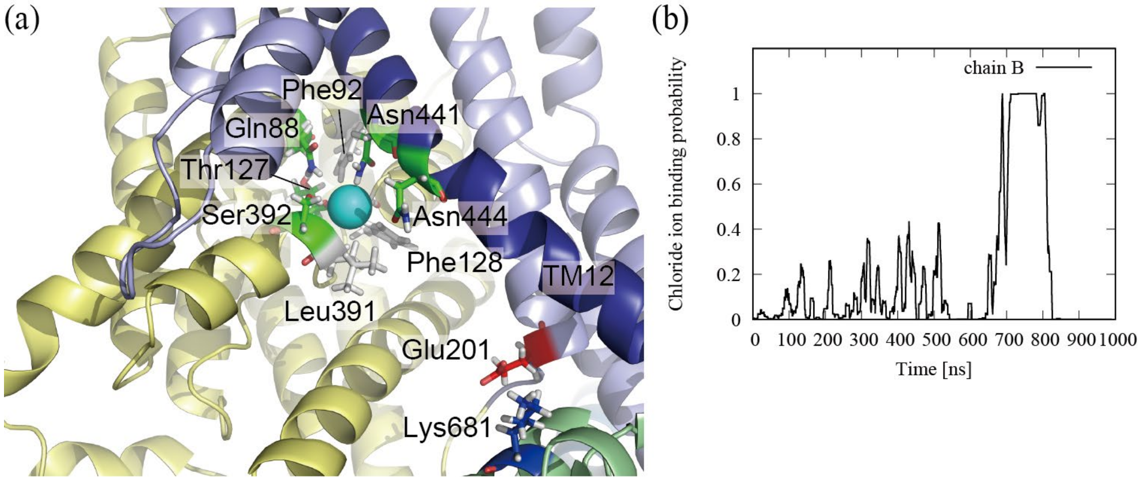

FIGURE S3. (a) Snapshot of ΔC-t1 simulation at 750 ns. The colors of the cartoon represent the same elements as in Figure 1, with the exception of the TM12 helix of the gate domain which is colored deep blue. Stick representations of basic (blue), acidic (red), polar (green), and hydrophobic (white) residues entering in contact with the chloride ion or forming salt bridges that trigger the domain motion of STAS are shown. Chloride ion stably bound to the binding site is shown as a sphere (cyan). (b) Time variation of the probability of chloride ion binding to the binding site of the chain B in ΔC-t1 simulation. Probabilities are window-averaged over a window width of 10 ns.

(a-1)

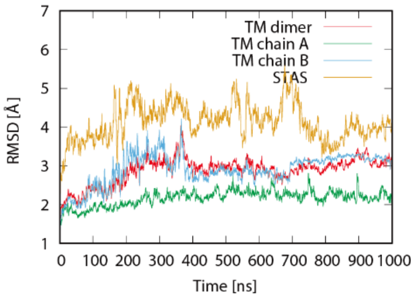

(a-2)

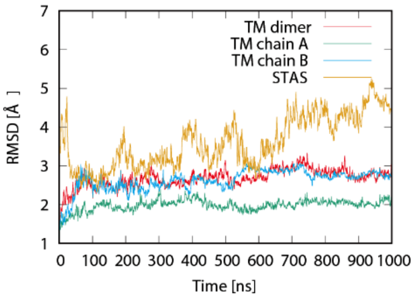

(b-1)

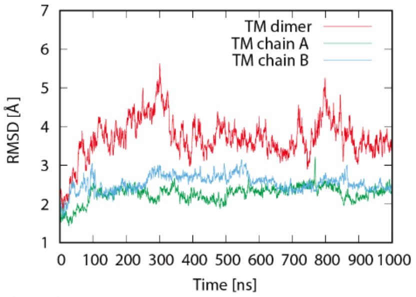

(b-2)

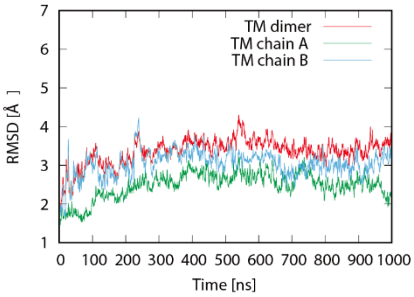

(c-1)

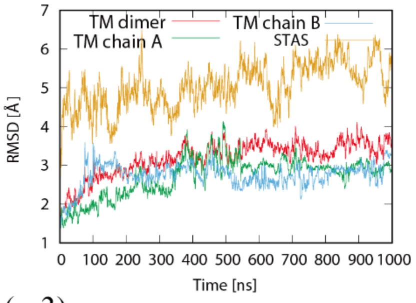

(c-2)

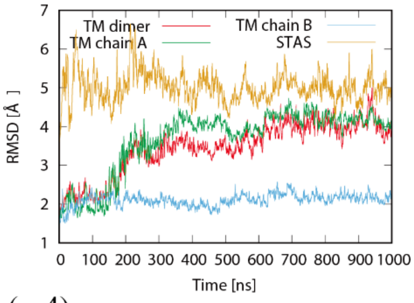

(c-3)

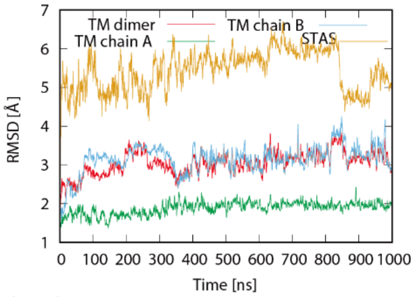

(c-4)

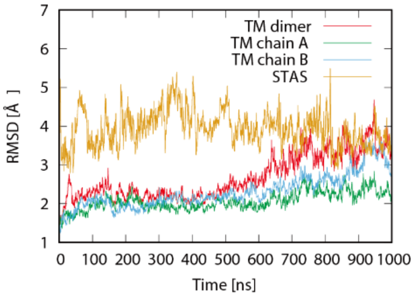

(c-5)

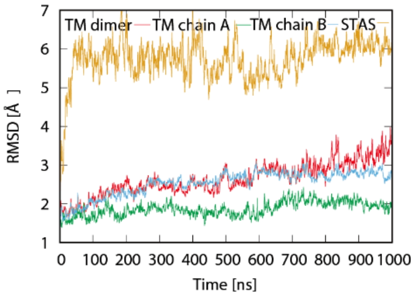

FIGURE S4. Time variation of RMSD from initial structure. “TM dimer” (red) represents the RMSD of the transmembrane domain of the dimer when superimposed in the transmembrane domains of both the chains A and B. “TM chain A” (green) represents the RMSD of the transmembrane domain of the chain A when superimposed in the transmembrane domain of the chain A only. “TM chain B” (cyan) represents the RMSD of chain B, which corresponds to that of chain A. “STAS” (orange) represents the RMSD of the STAS domain of the dimer when superimposed in the transmembrane domains of both the chains A and B. (a1-2) FL trajectory 1-2. (b1-2) ΔSTAS trajectory 1-2. (c1-5) ΔC trajectory 1-5.

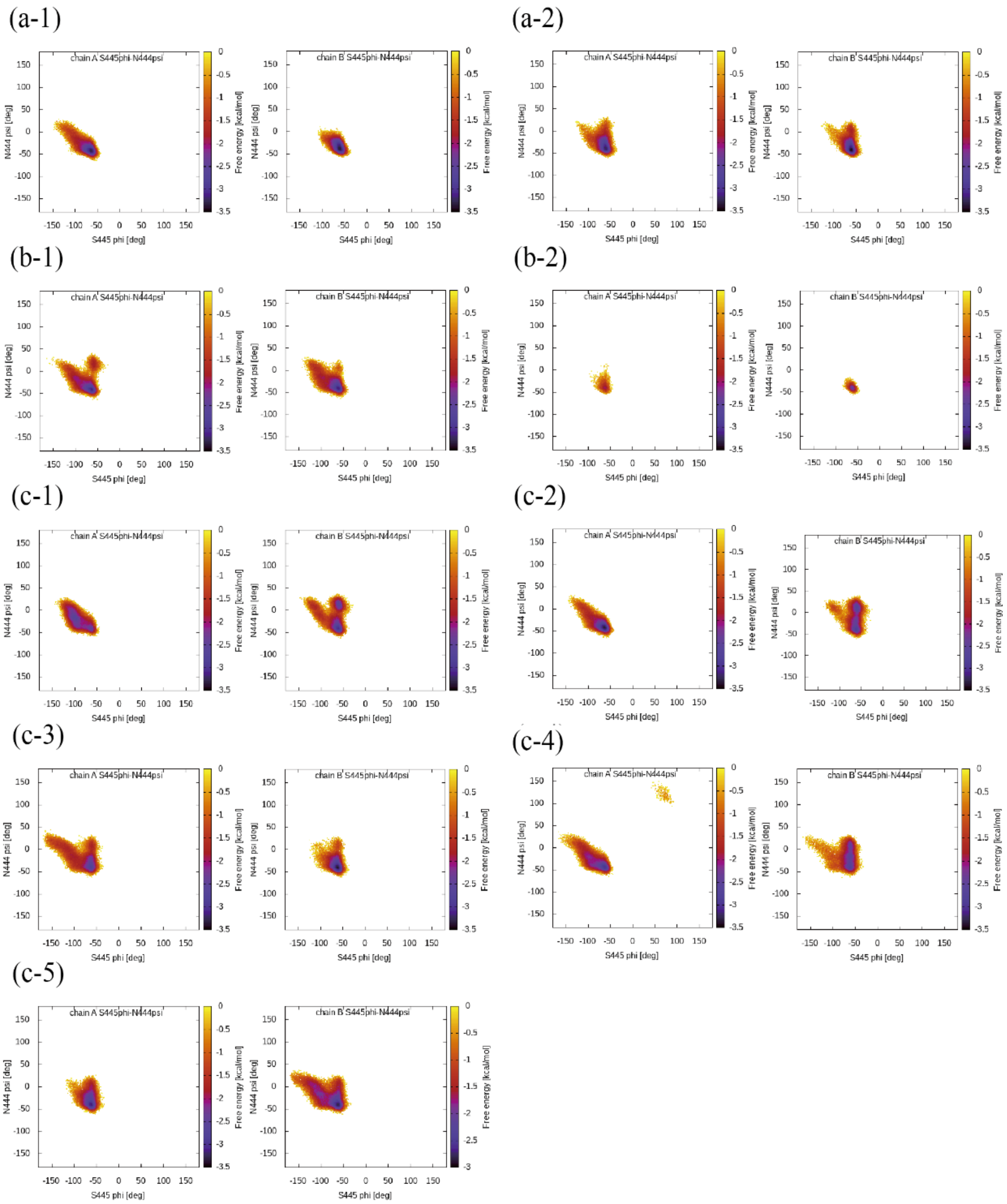

FIGURE S5.

Free energy landscape showing the correlation between the dihedral angles SER445φ and ASN444ψ. The free energy landscape for the chain A and the chain B is shown on the left and right for each trajectory, respectively. (a1-2) FL trajectory 1-2. (b1-2) ΔSTAS trajectory 1-2. (c1-5) ΔC trajectory 1-5.
